# Short-term efficacy of a computer-tailored physical activity intervention for prostate and colorectal cancer patients and survivors: a randomized controlled trial

**DOI:** 10.1186/s12966-018-0734-9

**Published:** 2018-10-30

**Authors:** Rianne Henrica Johanna Golsteijn, Catherine Bolman, Esmee Volders, Denise Astrid Peels, Hein de Vries, Lilian Lechner

**Affiliations:** 1 Department of Psychology and Educational Sciences, Open University of the Netherlands, PO Box 2960, 6401 DL Heerlen, The Netherlands; 2 Department of Health Promotion, Maastricht University, Maastricht, The Netherlands

**Keywords:** Prostate Cancer, Colorectal cancer, Physical activity, eHealth, Computer tailoring, Cancer survivorship, Fatigue, Quality of life, Depression, Accelerometer

## Abstract

**Background:**

Physical activity (PA) is beneficial in improving negative physical and psychological effects of cancer and cancer treatment, but adherence to PA guidelines is low. Computer-tailored PA interventions can reach large populations with little resources. They match with patients’ preference for home-based, unsupervised PA programs and are thus promising for the growing population of cancer survivors. The current study assessed the efficacy of a computer-tailored PA intervention in (four subgroups of) prostate and colorectal cancer survivors.

**Methods:**

Prostate and colorectal cancer patients and survivors were randomized to the OncoActive intervention group (*N* = 249), or a usual-care waiting-list control group (*N* = 229). OncoActive participants received a pedometer and computer-tailored PA advice, both Web-based via an interactive website and with printed materials. Minutes moderate-to-vigorous PA (MVPA) and days ≥30 min PA were assessed with an accelerometer (ActiGraph) at baseline and 6 months. Further, questionnaires were used to assess self-reported PA, fatigue, distress, and quality of life at baseline, 3 and 6 months. Differences between both groups were assessed using linear regression analyses (complete cases and intention-to-treat). In addition, efficacy in relation to age, gender, education, type of cancer, and time since treatment was examined.

**Results:**

Three months after baseline OncoActive participants significantly increased their self-reported PA (PA days: *d* = 0.46; MVPA: *d* = 0.23). Physical functioning (*d* = 0.23) and fatigue (*d* = − 0.21) also improved significantly after three months. Six months after baseline, self-reported PA (PA days: *d* = 0.51; MVPA: *d* = 0.37) and ActiGraph MVPA (*d* = 0.27) increased significantly, and ActiGraph days (*d* = 0.16) increased borderline significantly (*p* = .05; *d* = 0.16). Furthermore, OncoActive participants reported significantly improvements in physical functioning (*d* = 0.14), fatigue (*d* = − 0.23) and depression (*d* = − 0.32). Similar results were found for intention-to-treat analyses. Higher increases in PA were found for colorectal cancer participants at 3 months, and for medium and highly educated participants’ PA at 6 months. Health outcomes at 6 months were more prominent in colorectal cancer participants and in women.

**Conclusions:**

The OncoActive intervention was effective at increasing PA in prostate and colorectal cancer patients and survivors. Health-related effects were especially apparent in colorectal cancer participants. The intervention provides opportunities to accelerate cancer recovery. Long-term follow-up should examine further sustainability of these effects.

**Trial registration:**

The study was registered in the Dutch Trial Register (NTR4296) on October 17 2018.

**Electronic supplementary material:**

The online version of this article (10.1186/s12966-018-0734-9) contains supplementary material, which is available to authorized users.

## Background

Physical activity (PA) has numerous benefits for cancer patients and survivors. Positive effects have been reported for physical and psychological variables, such as cardiorespiratory fitness, muscle strength, fatigue, anxiety, depression, pain, physical functioning and thereby health-related quality of life (HRQoL) [1–8]. PA is also a preventive factor for other chronic diseases (e.g., cardiovascular disease, diabetes, osteoporosis) for which cancer survivors have an increased risk [5, 9–12]. Research has provided indications that PA is inversely associated with cancer-recurrence, development of secondary cancer and cancer mortality as well as overall mortality [2, 13, 14].

Despite all these benefits, the majority of cancer survivors do not meet PA guidelines, with self-reported rates ranging from 30 to 47% [15, 16], and accelerometer-measured rates being even lower [12, 17] (Golsteijn RHJ, Berendsen BAJ, Bolman C, Volders E, Lechner L: Cross-sectional and longitudinal measurement of physical activity in prostate and colorectal cancer patients and survivors: a validation and responsiveness study, submitted). Moreover, PA behavior declines during treatment, does not reach pre-treatment levels after completing treatment and is lower for cancer survivors in comparison to the general population [5, 18, 19]. In combination with cancer survivors’ need for healthy lifestyle information [20–22], this emphasizes the importance of developing effective programs to increase PA in cancer survivorship.

In 2012, over 14 million people were newly diagnosed with cancer worldwide [23] and this is expected to rise in the upcoming decades as a result of aging and advances in early detection [23, 24]. With advances in cancer treatment and early diagnosis, survival rates are improving and will result in an increasing population living after, and thus with the negative sequelae of cancer [25]. Thus, broad-reaching (i.e., non-face-to-face) PA programs, aimed at self-management, which can be provided in a cost-effective way, are especially important.

Evidence for the efficacy of PA interventions in improving cancer outcomes and treatment related side effects (e.g., fatigue, depression) and HRQoL [6, 10, 11, 26–29] is extensive, but mostly originates from interventions delivered face-to-face in a clinical or exercise setting. Such programs often report larger effect sizes compared to non-face-to-face interventions, but also come with considerable costs and it may be more difficult to implement them on a large scale. Few of such programs, however, examined effects with regard to PA behavior [29, 30]. Possibly, because they are not aimed at integrating PA into daily life and may lack real world application after ending the program [31, 32]. In addition, cancer survivors have a preference for home-based programs [33–35]. Hence, it is promising that reviews regarding interventions using non-face-to-face modalities (e.g., telephone, (tailored) print materials or internet) in general [36] and digital interventions explicitly [12] reported increases in PA and decreases in fatigue. Such interventions are much easier scalable to large settings and thus have the potential to reach large populations at relatively low costs.

Considering the advantages in terms of resources required, both for patients (time and travel) and care providers and the scalability, eHealth in particular, can provide important efforts in providing easily accessible PA interventions. Especially, since internet access and use are increasing in developed countries. In addition, perceived relevance can be increased by personalizing PA information through computer-tailoring, resulting in increased efficacy of such interventions [37, 38]. Nevertheless, we found that providing interventions only through the internet may exclude vulnerable sub-groups in a cancer population, such as those who are older, less educated, more fatigued or undergoing treatments [39]. Providing print-based tailored materials in addition to the online materials can be considered a solution to include these subgroups.

Accordingly, the OncoActive (OncoActief in Dutch) intervention was developed: a computer-tailored PA program providing PA advice online and with printed materials. Participants received automatically generated personalised feedback regarding PA and psychosocial determinants of PA at three time points. The content is aimed at the stimulating PA in daily life. To increase the probability of behavior change, the intervention is based on behavioral change theories [40–42] and on a demonstrated effective intervention for adults aged over fifty years [43, 44]. The aim of this study is to gain insight into the efficacy of the OncoActive intervention to increase PA.

Since the majority of evidence of PA interventions is currently based on trials conducted in breast cancer populations, there is need for interventions targeting other common cancer types [36] in order to improve cancer care in all cancer types. Therefore, the intervention was targeted at prostate and colorectal cancer, as these have a high incidence and good survival rates [45, 46]. By selecting only these two cancer types, we could better fine-tune the intervention to the specific PA needs and capabilities of the target group.

The purpose of the current study was to evaluate the efficacy of the OncoActive intervention at 3 months (during the intervention) and at 6 months (2 months after the intervention ended). As the intervention was aimed at increasing PA, the primary outcome is change in PA, assessed both for self-reported and accelerometer-measured PA. It was hypothesized that the intervention group would increase their PA more compared to the usual care group. As PA is also related to health-related outcomes of cancer patients and survivors [9] it was also hypothesized that the intervention group would decrease their fatigue, anxiety and depression and increase their physical functioning and overall HRQoL. Although the intervention was individually tailored, it might be that not all subgroups of participants respond similarly to the intervention. Therefore, we exploratively examined whether the efficacy differed for age, gender, education level, cancer type (i.e., prostate and colorectal) and time since treatment.

## Methods

### Study design

A parallel-group, randomized controlled trial (RCT), in which participants were allocated to either the OncoActive intervention group or a usual care waiting list control group (ratio 1:1) was conducted. Randomization was automatically performed by means of a digital randomizer after centralized registration of participants [47]. Due to the nature of the study, it was not possible or necessary to blind participants or the researchers. The RCT was approved by the Medical Ethics Committee of the Zuyderland hospital (NL47678.096.14) and is registered in the Dutch Trial Register (NTR4296). All participants provided written informed consent.

### Participants

Cancer patients and survivors (≥18 years) diagnosed with colorectal or prostate cancer could participate in the trial if they were undergoing treatment with a curative intent, or if they successfully completed primary treatment (surgery, chemotherapy or radiation) up to one year ago. They had to be at least 6 weeks post-surgery and there were no restrictions regarding patients undergoing hormonal therapy. Participants with severe medical, psychiatric or cognitive illness (e.g., Alzheimer’s disease, severe mobility limitations) were excluded from participation. Proficient Dutch reading and speaking skills were required for the questionnaires and for reading the tailored PA advice.

### Procedure

Over 12 months (in 2015 and 2016) prostate and colorectal cancer patients and survivors were recruited from the urology and/or oncology departments of seventeen hospitals throughout the Netherlands. Eligible participants were identified by hospital staff, verbally informed (either in person or by telephone) about the study, and invited to receive an information package. This written information was handed over or sent by mail. Additionally, cancer patients and survivors were invited via other channels (e.g., calls in local newspapers, on relevant websites, discussion groups, and flyers in hospitals). The researchers informed the interested participants, checked their eligibility, and provided them with an information package by mail.

The information package contained an information letter with a timeline of the study, an informed-consent form and a pre-paid return envelope. One postal reminder was sent after three weeks if there was no response to the information package. Cancer patients and survivors who agreed to participate were randomized into either the intervention group or the control group (usual care). Subsequently, baseline PA was assessed with an accelerometer (Actigraph GT3X-BT). Afterwards, participants received an online and paper-based questionnaire with the choice to fill out their preferred format. The intervention group received the OncoActive intervention after completing this baseline questionnaire (T0). Both groups filled-out follow-up questionnaires at three time points: 3 (T1), 6 (T2) and 12 (T3) months after baseline. Accelerometer PA measurements were conducted in the week prior to the T2 and T3 questionnaires. The usual care control group received the OncoActive intervention after completing the last measurement (T3).

### The OncoActive intervention

The OncoActive intervention is a computer-tailored intervention aimed at increasing awareness, initiation and maintenance of PA in prostate and colorectal cancer patients and survivors. The intervention was based on a demonstrated effective intervention to stimulate PA in adults over age fifty [43, 44] and adapted for prostate and colorectal cancer patients and survivors of all ages using the Intervention Mapping protocol [40]. The content was structured in line with behavioral change theories such as the I-Change Model [48–50], Social Cognitive Theory [51], Transtheoretical Model [52], Health Belief model [53], goal setting theories [54, 55], Health Action Process Approach [56], theories of self-regulation [57–59] and the Precaution Adoption Process Model [60].

Participants in the intervention group received computer-tailored PA advice at three time points (at baseline, after 2 months and after 3 months) both online on a secured website and on paper (by mail). The advice was generated automatically using a message library, questonnaire data and computer-based data-driven decision rules. The content of the first and second tailored advice was based on information gathered with the baseline questionnaire. Both the baseline (T0) and the second questionnaire (T1) provided input for the third tailored advice and allowed for the provision of ipsative feedback. The content of the advice was based on behavior change theories and targets pre-motivational constructs (e.g., awareness, knowledge), motivational constructs (e.g., self-efficacy, attitude, intrinsic motivation), and post-motivational constructs (e.g., goal setting, action and coping planning, self-regulation) [40, 61, 62]. In addition to the tailored advice, every participant received a pedometer and access to interactive content on the website (e.g., role model videos, home exercise instruction videos, a module for goal setting using a pedometer, the option to consult a physical therapist and additional information). A more detailed description of the intervention content can be found elsewhere [40]. Use of the advice was examined through self-report. Percentages of participants reporting not having read any advice ranged from 0.6 to 6.1% time point [39].

### Measurements

As it was the main goal of the OncoActive intervention to improve PA, the primary outcome is PA behavior. Health-outcomes including fatigue, distress and HRQoL are examined as secondary outcomes.

#### PA outcomes

As PA comprises a complex behavior consisting of type of activity, duration, frequency, and intensity, PA was measured both with questionnaires and accelerometers [63]. Although self-report questionnaires are known for their overestimation of MVPA, they measure different constructs than accelerometers [64] (Golsteijn RHJ, Berendsen BAJ, Bolman C, Volders E, Lechner L: Cross-sectional and longitudinal measurement of physical activity in prostate and colorectal cancer patients and survivors: a validation and responsiveness study, submitted). Therefore, a combination of both might present the most complete insight in PA.

Self-reported PA was measured using the validated Short Questionnaire to Assess Health Enhancing Physical Activity (SQUASH) [65], assessing activities regarding commuting, household, occupation, and leisure time. Total minutes of PA were classified into light (metabolic equivalent [MET] < 3.0), moderate (MET 3.0–5.9), and vigorous (MET > 6) [66]. Minutes of moderate to vigorous PA (MVPA) were calculated by adding up total time in moderate and vigorous PA. Participants with extreme values (i.e., > 6720 min PA/week), were excluded in accordance with the SQUASH scoring manual. The SQUASH questionnaire also contains a single-item measure assessing the number of days in the past week, on which one is at least moderately physically active for 30 min or more. The SQUASH questionnaire has reasonable reliability (ρ = .58) and validity against an accelerometer (ρ = .45) [65].

Additionally, PA was measured using the ActiGraph GT3X-BT (ActiGraph, Pensacola, FL). Participants wore the accelerometer on an elastic belt on their right hip for 7 days. Data were downloaded and analyzed using ActiLife software (ActiGraph, Pensacola, FL). Measurements were considered valid if there were at least 4 days with at least 10 h of wear time per day [67]. Non-wear periods were excluded from the analyses and were identified in accordance with Choi et al. [68]: intervals of at least 90 consecutive min of zero counts with allowance of a maximum of 2 min of nonzero counts during a non-wear interval. MVPA was calculated using 3 axes based on 60 s epochs.Freedson-VM cut-off points (developed by Sasaki et al. [69]) and the cut-off point developed by Aguilar-Fariaz et al. [70] to distinguish between light, moderate and vigorous PA.

#### Health-related outcomes

Health-related outcomes assessed in the current study included fatigue, distress and HRQoL. Fatigue was measured with the Checklist Individual Strength (CIS) [71]. The questionnaire consists of 20 items which are scored on a scale from 1 to 7, resulting in a total score ranging from 20 to 140 (alpha = .919), with a higher score indicating more fatigue. The CIS contains 4 subscales (subjective fatigue, concentration, motivation, and activity), but the total score, which was used in the current study, provides an overall indication of fatigue [72]. Missing items were imputed with the mean of the subscale and were limited to 1 item per subscale.

Distress was assessed with the 14-item Hospital Anxiety and Depression Scale (HADS) [73, 74]. The questionnaire consists of two scales, each one comprising 7 items with a 4-point scale, measuring anxiety (alpha = .799) and depression (alpha = .798.). Scale scores range from 0 to 21. A maximum of 1 missing item per scale was imputed with the mean of the respective subscale [75].

HRQoL was measured with the European Organization for Research and Treatment of Cancer Quality of Life Questionnaire-C30 (EORTC QLQ-C30) [76]. In the current study, we assessed global health status (2 items (alpha = .837) on a 7-point scale) and physical functioning (4 item (alpha = .683) on a 5-point scale) as these have the strongest relation with PA [77]. Scores were converted to scores ranging from 0 to 100, with a high score indicating a high HRQoL.

#### Other relevant measures

Demographic and cancer-related characteristics including age, gender, body mass index (BMI), educational level, type of cancer, type of treatment (e.g., surgery, chemo therapy, radiotherapy and hormonal treatment), treatment phase (during or after) and elapsed time since final treatment were assessed in the baseline questionnaire. Educational level was categorized into low (i.e., primary, basic vocational, or lower general school), moderate (i.e., medium vocational school, higher general secondary education, and preparatory academic education), or high (i.e., higher vocational school or university level) according to the Dutch educational system. Participants were classified as being overweight (BMI > 24.9 kg/m^2^) or not. Cancer-related characteristics included type of cancer, which was either prostate or colorectal in the current study, and date of their last treatment. In addition, the presence of a chronic disease (yes or no) and the intention to be physically active (3 items on a scale from 1 to 10 (alpha =0.91), [62, 78]) were assessed at baseline.

#### Timing of assessments

PA assessments with the ActiGraph were carried out at baseline and 6 months thereafter. Both self-reported PA and health-related outcomes were assessed using questionnaires at baseline (T0), 3 months (T1) and 6 months (T2; 2 months after the end of the intervention). At baseline all outcome measures were assessed. In the T1 questionnaire, which was conducted during the intervention period, we tried to limit the burden for participants by including only questions which were necessary to generate computer tailored advice for the intervention group (although the control group completed the same questionnaire). These included the SQUASH (self-reported MVPA & days ≥30 min PA), CIS (fatigue), and the physical functioning and general HRQoL subscales of the EORTC QLQ-C30. At T2 in addition, accelerometer-measured PA (ActiGraph) and the HADS (anxiety and depression) were assessed.

### Sample size

Sample size calculations were based on the PA outcomes of predecessors of the intervention in adults aged fifty years or older [43, 44]. These studies found an effect size of 0.3 with regard to PA (primary outcome) and effects were assumed to be comparable in cancer patients and survivors. Power calculations showed that approximately 300 participants were needed in total for the current study based on this effect size, a power of .80 with an alpha of .05 and a correction for multilevel analyses (intracluster correlation coefficient (ICC) = .005, design effect = 1.15). Drop-out was expected to be around 30% during the study [27, 43, 44], thus in total 428 participants were needed for enrollment at baseline.

### Statistical analyses

Baseline differences regarding demographics, cancer-related, health-related, and PA-related characteristics between both conditions were assessed with independent t-tests and chi-square test. Group assignment, demographics, cancer and health-related characteristics and baseline values of the outcome measures were assessed as predictors of dropout at 3 and 6 months using logistic regression.

Multilevel linear regressions (linear mixed models) were conducted to analyze the results. With patients originating from different hospitals, it was expected that their data was clustered. In order to adjust for this clustering, we applied multilevel linear regression with participants nested in hospitals. However, these analyses revealed that the ICC was almost zero (i.e., 1.09e^− 13^) and correction for clustering was not necessary. In addition, with multiple timepoints there is also a possibility of interdependence between the measurements within a person. Therefore, time, group and the interaction between time and group (to study differences between both groups over time) were added to the mixed models providing the opportunity to assess intervention efficacy over time. The models were fitted using the maximum likelihood procedure and an independent covariance structure. For all analyses age, gender, educational level, type of cancer, treatment phase, time since last treatment, BMI, comorbidity, PA intention and the baseline values of the outcome measure were added as covariates. Raw means of primary and secondary outcomes at all time points were presented. In addition Cohen’s d effect sizes were calculated for all outcomes, with effect sizes of 0.20, 0.50, and 0.80 indicating small, medium, and large effects respectively [79].

Although drop out was limited, we applied intention-to-treat (ITT) analyses in addition to the complete case analysis. With multiple imputations (20 times) missing data at 3 and 6 months was imputed including all covariates, the independent variable, and the outcome measure as predictors.

Intervention effectiveness was also assessed in different subgroups of participants. Therefore, interaction terms for age, gender, educational level, type of cancer and time since treatment were added to the regression. To test the moderation effects, 3 and 6 month measurements were analyzed separately. When an interaction term was significant, subgroup effects were examined. Since interaction terms have less power, the significance levels were set to *p* < .10 [80]. Significance levels for other analyses were set to *p* < .05. All analyses were conducted using STATA version 13.1.

## Results

### Study population

**Fig. 1:**
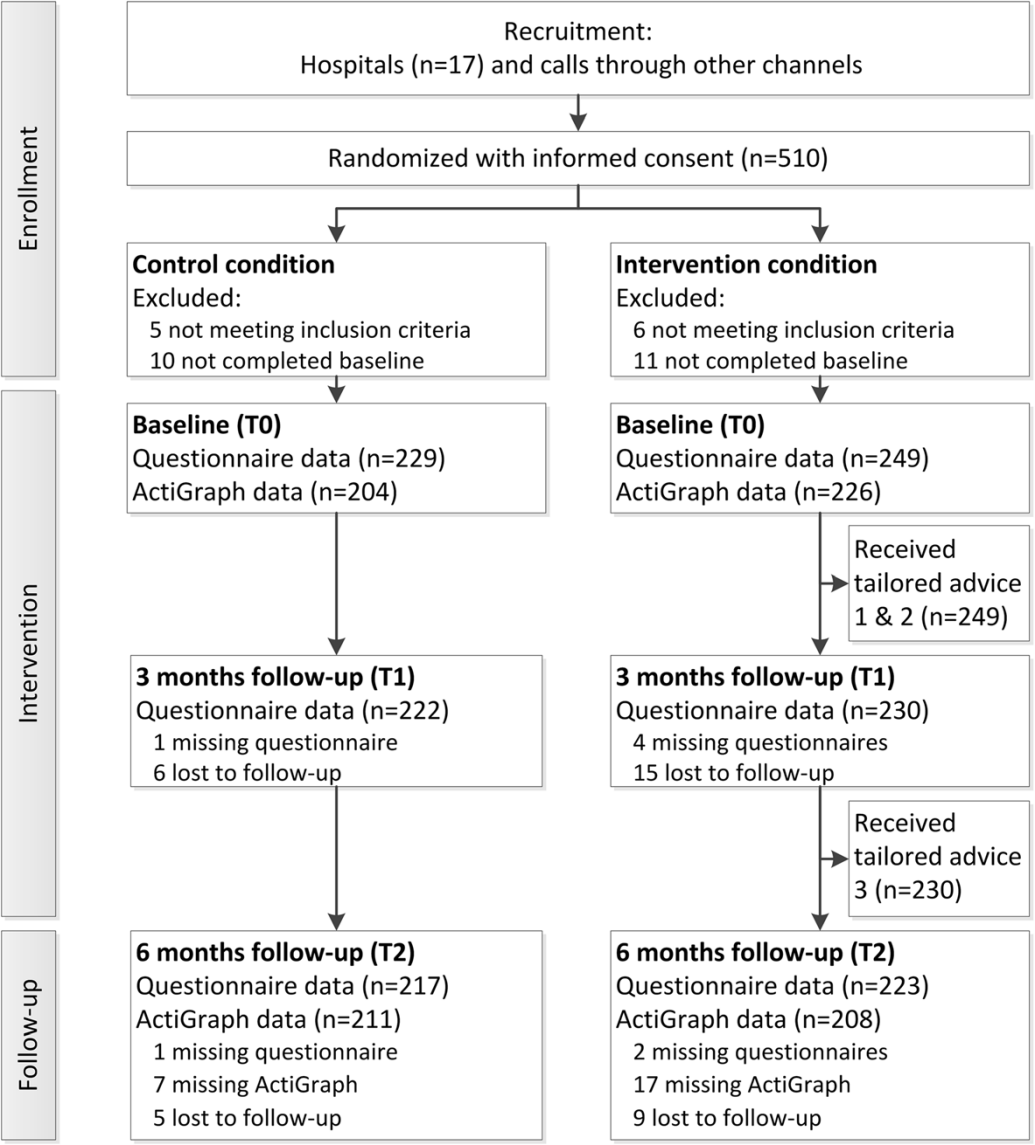
Flow diagram of the study

An overview of the number of participants who are enrolled in the intervention and participated in the 3 and 6 months follow-up measurements is provided in Fig. 1.

Drop-out rates were very low with 4.4% (21/478) of the participants dropping out at the 3 months follow-up and 7.3% (35/478) dropping out at the 6 months follow-up. Attrition analyses showed that at 3 months participants in the intervention group (B = 1.43, 95%CI = 0.02–2.84, *p* = .047) and participants with a lower intention to be physically active (B = 0.53, 95%CI = 0.01–1.05, p = .047) were more likely to drop out of the study. At 6 months, colorectal cancer patients were more likely to dropout (B = 1.05, 95%CI = 0.06–2.04, *p* = .034).

Participant characteristics of both groups are shown in Table 1. The mean age was 66.5, the majority of the participants were male (87%) and the proportion of prostate cancer was 61% compared to 39% colorectal cancer. The control group and intervention group differed on the depression score, with a significantly higher baseline score for the intervention group (*p* = .01).

**Table 1:** Baseline participant characteristics of the intervention group and the control group

	Intervention group (OncoActive, *n* = 249)	Control group (Usual care, *n* = 229)	*P* value
Demographic characteristics			
Age in years, mean (SD)	66.55 (7.07)	66.38 (8.21)	.81
Gender, n (%)			.20
Male	212 (85.1)	204 (89.1)	
Female	37 (14.9)	25 (10.9)	
Education, n (%)			.15
Low	109 (44.0)	114 (50.0)	
Middle	70 (28.2)	47 (20.6)	
High	69 (27.8)	67 (29.4)	
Cancer related characteristics			
Type of cancer, n (%)			.34
Prostate	149 (59.8)	143 (62.5)	
Colorectal	100 (40.2)	86 (37.5)	
Treatment phase, n (%)			.42
During treatment	19 (7.6)	14 (6.1)	
After treatment	230 (92.4)	215 (93.9)	
Time since last treatment in months, mean (SD)	5.64 (3.84)	5.17 (3.49	.16
Type or treatment, n (%)			
Surgery	186 (81.2)	192 (77.1)	.27
Chemo	41 (17.9)	44 (17.7)	.95
Radiotherapy	63 (27.5)	80 (32.1)	.27
Hormonal treatment	8 (3.5)	10 (4.0)	.76
Health related characteristics			
BMI, mean (SD)	26.39 (3.38)	26.74 (4.41)	.32
Comorbidities yes, n (%)	87 (35.2)	86 (38.2)	.46
Fatigue, mean (SD)	58.95 (23.31)	57.54 (24.25)	.52
Anxiety, mean (SD)	3.75 (3.22)	3.37 (2.95)	.18
Depression, mean (SD)	3.54 (3.54)	2.80 (2.91)	*.01*
General HRQoL, mean (SD)	80.01 (16.81)	82.06 (14.15)	.15
Physical Functioning, mean (SD)	86.57 (14.39)	86.58 (14.80)	.99
PA characteristics			
MVPA SQUASH, mean (SD)	798 (721)	873 (764)	.27
MVPA ActiGraph, mean (SD)	271 (211)	293 (230)	.30
Days ≥30 min PA SQUASH, mean (SD)	3.67 (2.05)	3.86 (2.07)	.34
Days ≥30 min PA ActiGraph, mean (SD)	3.23 (2.46)	3.38 (2.38)	.52
PA intention, mean (SD)	7.61 (1.35)	7.74 (1.48)	.32

### Intervention effects at 3 month follow-up

Raw means at baseline and at 3 month follow-up (still during the intervention period) for both conditions are shown in Table 2. These raw scores indicated improvements in PA, fatigue and physical functioning, but not in general HRQoL. To test for significance additional statistical analyses were performed. The results are shown in Table 3. Participants in the OncoActive group improved their PA significantly in terms of both MVPA (B = 133.55, *p* = .04) and days with at least 30 min of PA (B = 0.86, *p* < .001). With regard to the secondary outcomes, we found decreased fatigue (B = − 3.57, *p* = .02) and improved physical functioning (B = 2.61, *p* = .003) for participants of the OncoActive intervention. No significant differences were found with regard to overall HRQoL (B = 0.18, *p* = .82). ITT analyses showed similar results for all outcomes.

**Table 2:** Raw means of primary and secondary outcomes at all time points

	Intervention group (OncoActive)						Control group (Usual Care)					
	T0		T1		T2		T0		T1		T2	
	N	Mean (SD)	N	Mean (SD)	N	Mean (SD)	N	Mean (SD)	N	Mean (SD)	N	Mean (SD)
Primary outcomes												
SQUASH MVPA	246	780 (721)	230	1060 (771)	222	1145 (883)	229	873 (764)	221	962 (833)	213	943 (769)
SQUASH Days ≥30 min PA	246	3.70 (2.06)	226	4.81 (1.89)	218	5.18 (1.65)	226	3.86 (2.07)	222	4.02 (2.06)	210	4.31 (1.93)
ActiGraph MVPA^a^	226	271 (211)	–	–	208	331 (234)	204	293 (229)	–	–	211	301 (219)
ActiGraph Days ≥30 min PA^a^	226	3.35 (2.54)	–	–	208	3.96 (2.38)	204	3.46 (2.40)	–	–	211	3.71 (2.38)
Secondary outcomes												
Fatigue	241	58.9 (23.3)	227	54.5 (22.5)	221	51.6 (23.9)	223	57.5 (24.3)	217	56.7 (23.4)	214	55.1 (23.7)
Physical functioning	246	86.6 (14.0)	230	89.6 (13.0)	223	89.7 (13.6)	229	86.6 (14.8)	222	87.4 (14.1)	216	88.4 (13.0)
General HRQoL	246	80.0 (16.8)	229	79.8 (16.3)	223	83.8 (15.6)	229	82.1 (14.2)	222	80.7 (14.8)	216	83.7 (13.7)
Anxiety^a^	248	3.75 (3.21)	–	–	223	3.52 (3.39)	227	3.37 (2.95)	–	–	216	3.49 (3.17)
Depression^a^	248	3.54 (3.54)	–	–	223	3.09 (3.34)	227	2.80 (2.91)	–	–	216	3.31 (3.08)

^a^ Outcomes assessed only at T2 measurement to limit participant burden at T1; see methods section for explanation

**Table 3:** Outcomes at 3 months follow-up

	Complete case analyses				Intention to treat analyses		
	N	B (95% CI)	p	ES^a^	N	B (95% CI)	p
Primary outcomes							
MVPA SQUASH	437	133.55 (3.70–263.40)	.04	0.23	462	139 (9.41–268.97)	.04
Days ≥30 min PA SQUASH	433	0.86 (0.55–1.18)	<.001	0.46	461	0.82 (0.52–1.13)	<.001
Secondary outcomes							
Fatigue	425	−3.57 (−6.68 – − 0.46)	.02	− 0.21	453	−3.66 (− 6.78 – − 0.54)	.02
Physical functioning	440	2.61 (0.86–4.36)	.003	0.23	464	2.33 (0.54–4.12)	.01
General HRQoL	439	0.18 (−2.19–2.55)	.88	0.09	464	−0.02 (− 2.39–2.35)	.99

^a^ Based on mean difference between T0 and T1

### Intervention effects at 6 month follow-up

Raw means for the 6 month follow-up assessment are shown in Table 2 indicating further improvements in PA, fatigue and physical functioning. Depression scores also improved in the intervention group. Further statistical analyses were performed to examine the efficacy after finishing the intervention (6 month follow-up) (Table 4). Results indicate significant improvements in PA assessed through the SQUASH questionnaire (MVPA: B = 267.17, *p* < .001; Days ≥30 min PA: B = 0.98, p < .001). ActiGraph assessed MVPA also increased significantly (MVPA: B = 44.60, *p* = .006), whereas the increase in ActiGraph assessed days ≥30 min PA was borderline significant (B = 0.38, *p* = .05).

**Table 4:** Outcomes at 6 months follow-up

	Complete case analyses				Intention to treat analyses		
	N	B (95% CI)	p	ES^a^	N	B (95% CI)	p
Primary outcomes							
MVPA							
SQUASH	421	267.17 (135.12–399.22)	<.001	0.37	462	275 (141.14–408.59)	<.001
ActiGraph	373	44.60 (12.57–76.63)	.006	0.27	420	45.9 (13.51–78.30)	.006
Days ≥30 min PA							
SQUASH	415	0.98 (0.66–1.30)	<.001	0.51	461	0.93 (0.62–1.24)	<.001
ActiGraph	373	0.38 (− 0.01–0.77)	.05	0.16	420	0.37 (− 0.01–0.75)	.06
Secondary outcomes							
Fatigue	416	−4.16 (−7.30 – − 1.02)	.009	− 0.23	453	− 3.88 (− 7.02 – − 0.74)	.015
Physical functioning	427	1.86 (0.09–3.63)	.04	0.14	464	1.31 (− 0.48–3.10)	.15
General HRQoL	427	1.09 (−1.30–3.49)	.37	0.13	464	0.69 (−1.71–3.06)	.58
Anxiety	427	−0.14 (− 0.59–0.30)	.54	−0.11	464	−0.15 (− 0.60–0.29)	.51
Depression	427	−0.64 (− 1.09 – - 0.19)	.005	−0.32	464	−0.61 (− 1.06 – − 0.16)	.008

^a^ Based on mean difference between T0 and T2

There were also significant improvements in health-related outcomes. In comparison to the control group, a decrease in fatigue (B = − 4.16, *p* = .009) and depression (B = − 0.64, *p* = .005), and an improvement in physical functioning (B = 1.86, *p* = .04) were observed for the OncoActive group. No significant differences were found for anxiety (B = 0.14, *p* = .54) and overall HRQoL (B = 1.09, *p* = .37). Similar results were found in the ITT analyses, except for physical functioning which did not improve significantly in the ITT analysis.

### Moderation of effects

To further explore the efficacy of the intervention, analyses for subgroups were performed. These exploratory analyses showed that the intervention effect on PA was moderated by education level and type of cancer. The 3 month effect on MVPA as reported by the SQUASH questionnaire was moderated by cancer type (*p* = .02): The intervention was effective at increasing PA in colorectal cancer participants (B = 355.23, *p* = .001, ES = 0.53), but not in prostate cancer participants (B = 20.33, *p* = .81, ES = 0.07). MVPA assessed with the ActiGraph at 6 months was moderated by education level (*p* = .06). OncoActive resulted in a significant increase in MVPA in participants with a medium education level (B = 106.85, p = .001, ES = 0.59), in a borderline significant increase for highly educated participants (B = 56.33, p = .06, ES = 0.42) and no increase for those with a low education (B = − 0.11, *p* = .99, ES = .03).

Health outcomes were moderated by gender and type of cancer. At the 3 month follow-up fatigue was moderated by type of cancer (*p* = .04). Fatigue levels of colorectal cancer participants significantly decreased (B = − 6.88, *p* = .02, ES = − 0.31), whereas no significant decrease was found for prostate cancer participants (B = − 1.69, *p* = .34, ES = − 0.14). Physical functioning at 3 months was also moderated by type of cancer (*p* = .003). Again, significant improvements were found for colorectal cancer participants (B = 6.32, *p* < .001, ES = 0.45), but not for prostate cancer participants (B = 0.77, *p* = .45, ES = 0.06).

At 6 month follow-up, fatigue was moderated by gender (p = .02). OncoActive resulted in a significant decrease in fatigue in women (B = − 12.70, *p* = .007, ES = − 0.76), but not in men (B = − 2.14, *p* = .21, ES = − 0.15). Type of cancer moderated the effects on depression (*p* = .07) and physical functioning (*p* = .03). Depression decreased significantly in colorectal cancer participants (B = − 1.17, *p* = .004, ES = − 0.37), but not in prostate cancer participants (B = − 0.44, *p* = .10, ES = − 0.30). Similar results were found for physical functioning, with significant improvements in colorectal cancer participants (B = 4.27, *p* = .01, ES = 0.35), but not in prostate cancer participants (B = 0.31, *p* = .73, ES = − 0.004).

## Discussion

The current study assessed the efficacy of the computer-tailored OncoActive intervention at increasing PA and in improving fatigue, HRQoL and distress (i.e., anxiety and depression) in prostate and colorectal. In addition, efficacy in specific subgroups of cancer patients was explored.

### PA outcomes

The hypothesis that the intervention group would increase their PA, was confirmed by the finding that OncoActive participants increased both in MVPA and in the number of days on which they were physically active for at least 30 min. As mentioned, PA was measured both with an accelerometer and with a self-report questionnaire as both measures have strengths and weaknesses [63] (Golsteijn RHJ, Berendsen BAJ, Bolman C, Volders E, Lechner L: Cross-sectional and longitudinal measurement of physical activity in prostate and colorectal cancer patients and survivors: a validation and responsiveness study, submitted). With regard to MVPA, it was noted that although the absolute increase was substantially higher for self-reported PA compared to PA assessed by the ActiGraph, findings were clearly in the same direction (Additional file 1). Absolute increases of 280 (3 months) and 365 (6 months) minutes MVPA per week for self-reported PA and 60 min (6 months) MVPA for ActiGraph PA (based on raw scores; Table 2) were found in the intervention group. In comparison increases of 89, 70, and 8 min respectively were found in the control group. As a meta-analysis regarding digital PA interventions in cancer patients found an average increase of 40 min MVPA based on self-report PA [12], the OncoActive intervention thus seems to be highly effective in increasing PA. Intervention studies using accelerometer-measured PA as outcome variables are lacking [12], therefore it is recommended to include them in future studies.

Several explanations can be provided for the substantial differences between both PA measures. Self-report questionnaires are known for their probability of over-reporting, whereas accelerometers are not able to measure certain activities properly (e.g., swimming, cycling, upper body movement), and they cannot assess the type of PA (e.g., leisure time PA, PA for transportation, occupational PA) [81]. In addition, ActiGraph outcomes regarding light, moderate and vigorous PA are based on cut-points developed for healthy adults [69, 82, 83]. However, as cancer patients and survivors may have decreased physical fitness they possibly perceive certain activities as moderately intensive, whereas the Actigraph classifies them as light activities (Golsteijn RHJ, Berendsen BAJ, Bolman C, Volders E, Lechner L: Cross-sectional and longitudinal measurement of physical activity in prostate and colorectal cancer patients and survivors: a validation and responsiveness study, submitted).

Effect sizes for MVPA were small (0.23–0.37), yet comparable to other studies. Effect sizes for the Active Plus intervention (healthy adults aged over fifty), from which the OncoActive intervention was developed, also ranged from 0.23 to 0.35 [84]. Meta-analyses regarding computer-tailored and web-based PA interventions for healthy and diseased adult populations found average Cohen’s *d* of 0.14 [85] and Hedge’s *g* of 0.16 [86]. Kanera et al. [26] also found a comparable Cohen’s *d* of 0.25 for moderate PA in a multiple lifestyle eHealth intervention for cancer survivors. A review regarding broad-reach modality PA interventions for cancer survivors found effect sizes for MVPA outcomes in the same range as the current study [36].

Besides MVPA, days on which participants were physically active for at least 30 min were also examined. Significant increases were found for self-report at 3 and 6 months, but the ActiGraph measured outcome at 6 months was only borderline significant (*p* = .05). Again, this can possibly be explained by the nature of the two measurements. Besides the earlier mentioned discrepancies in classifying light and moderate intensity PA, it might also be difficult to exactly estimate time in self-report. If someone is physically active for 25 min, one might experience this as being physically active for at least 30 min and thus report it accordingly. Since the ActiGraph measures and classifies every single minute, such a day would not be included in the ActiGraph measure for days with ≥30 min PA, resulting in a discrepancy between both measures.

### Health-related outcomes

Besides being effective in increasing PA, it was also hypothesized that the OncoActive intervention would improve health-related outcomes such as fatigue, HRQoL and distress. This hypothesis was partially confirmed by the findings of the current study as significant improvements were found for fatigue, depression and physical functioning, but not for anxiety and general HRQoL.

Fatigue levels of OncoActive participants decreased significantly during the intervention period and decreased even further during the second part of the intervention period, resulting in significantly less fatigue two months after the last tailored advice. This is in accordance with findings in several systematic reviews on health outcomes such as fatigue among cancer patients [3, 4, 87]. However, most of the studies in these reviews are supervised exercise trials with a health outcome like fatigue as the primary outcome measure. Such trials are often aimed at improving health outcomes instead of improving PA [88]. The main aim of the current study, which would be classified as a behavior change trial by Courneya [88], was to improve PA, with fatigue and other health outcomes being secondary outcomes. As a meta-analysis regarding digital behavior change interventions in cancer survivors only found a non-significant trend towards decreased fatigue [12], it is very promising that the OncoActive intervention was able to improve fatigue.

Reviews from Mishra et al. [3, 4] and Sweegers et al. [77] found that exercise interventions are able to establish significant benefits with regard to general HRQoL. However, for the OncoActive intervention no improvement in overall HRQoL was observed. Similarly, Roberts et al. [12] also did not find improvements in HRQoL for digital behavior change interventions. A possible explanation for not finding any effects regarding HRQoL could be the high baseline scores of our study population. Both the intervention group and the control group had general HRQoL baseline scores above 80 (on a 0–100 scale). With such high baseline scores, it may be difficult to improve further. Also, baseline scores in our study were higher than in other studies that did find significant improvements in HRQoL [89].

Nevertheless, physical functioning did improve significantly in OncoActive participants both during and after the intervention period, indicating that OncoActive may accelerate cancer recovery, especially since the effects were more apparent during the first 3 months of the intervention. The absence of a similar improvement for 3 to 6 months after baseline, may be due to ceiling effects, as the levels of physical functioning were already high at the 3 month measurement (i.e., 89.6 on a scale from 0 to 100 in the intervention group). A systematic review also reported improvements in physical functioning through home- and community-based PA programs with effect sizes ranging from .17 to .45, with larger effect sizes for community-based programs with group meetings [90]. Thus, it can be concluded that effect sizes found for the OncoActive intervention (0.23 at 3 months and 0.14 at 6 months) are in the same range of home-based PA programs without group meetings.

Findings in the literature regarding anxiety and depression are mixed. Some reviews and studies reported improvements in anxiety, whereas others reported improvements in depression [3, 4, 12, 91]. For the OncoActive intervention, no significant improvements were found regarding anxiety. For depression we found a significant improvement in the intervention group. However, even though we corrected for baseline differences in depression symptoms, this finding should be interpreted with caution as the intervention group had a significant higher depression score at baseline. As a result, regression to the mean might have influenced our results.

In general, it is promising that a computer-tailored intervention, which can be provided to a relatively large population at relatively low costs, is able to improve treatment-related side effects and thereby cancer recovery. Future research should focus on reaching and assessing long term maintenance of intervention effects.

### Efficacy in subgroups

In the current study effects in specific subgroups of cancer patients and survivors were studied exploratory. Results showed that during the first part of the intervention, PA only increased in colorectal cancer participants. However, two months after completing the intervention, OncoActive was equally effective in increasing PA for both cancer types. As the intervention is tailored to cancer type, future research could extend the intervention to other types of cancer.

The explorative moderation analyses also showed that the intervention was effective in increasing PA in those with a medium and high education level, but not in those with a low education level. Possibly, receiving information about behavior change may have decreased lower educated participants’ self-efficacy to be able to change their PA and may have resulted in perceiving the recommendations as less feasible [92]. Another explanation can be found in the structure of the OncoActive intervention. Intervention materials were provided both print- and web-based alongside each other. In a previous study regarding the OncoActive intervention, a lower educational level was associated with a lower probability of using Web-based materials [39]. Although this previous study also showed that tailored advice was read by most of them, those with a lower education may have had a less comprehensive experience with the intervention as they may not have viewed video incorporated in the web-based tailored PA advice or used other interactive (web-based) components of the intervention. For future studies implementation adaptations, like less written texts, should be made to improve efficacy in cancer patients and survivors with a lower educational level.

With regard to health-related outcomes, it was noted that the intervention in general was more effective for colorectal cancer participants than for prostate cancer participants. At 3 months effects on fatigue and physical functioning, and at 6 months effects on depression and physical functioning were stronger among colorectal cancer participants. In addition, at 6 months we found a larger effect on fatigue for women compared to men. Since, women can only be diagnosed with colorectal cancer this might also be linked to cancer type. As health effects do take some time to occur, a possible explanation for better health effects in colorectal cancer participants might be that PA did not increase in the first 3 months of the intervention in prostate cancer participants. Another explanation may be the fact additional in depth analyses showed that raw baseline scores for prostate cancer participants were higher in comparison to colorectal cancer participants, resulting in less room for improvement. Nevertheless, since PA did improve significantly after the intervention, evaluation at 12 month follow-up should prove whether there will be further improvements in health-related outcomes in prostate cancer participants on the longer term. Furthermore, if the increase in PA can be maintained, eventually cancer survivors may develop a healthier lifestyle [32] and possibly benefit from improved survival [13, 14].

### Strengths and limitations

The current study has a strong research design (RCT) in which both self-reported and accelerometer-measured PA were assessed. In addition, a very low dropout rate of only 7% was observed in the current study. Such a low dropout rate is exceptional in digital interventions [93] and in the same range of supervised exercise programs [89, 94, 95]. Although promising results were found regarding the efficacy of the OncoActive intervention, there are also some limitations that should be acknowledged.

In the current study, the proportion of participants who had adjuvant treatment is relatively small. This can partly be attributed to the current treatment preferences for the types of cancer in the target group. Currently prostate cancer is most often treated with surgery or brachy therapy, which might be less invasive than external radiotherapy. Furthermore, in 2014 a screening program for colorectal cancer was introduced in the Netherlands. Due to this increased early detection, patients may be diagnosed in the early stage of the disease. Consequently, there are fewer patients that need to undergo adjuvant chemotherapy and thus experience fewer treatment-related side effects. As a result, the effect found in the current study may not be representative for patients undergoing more burdensome (adjuvant) treatments.

Although participant dropout was very low, it was related to cancer type, intention to be physically active and group assignment. Although this may affect findings, it is expected that the influence of selective dropout is negligible due to the very low dropout numbers.

With regard to the health-related outcomes, it should be noted that these analyses may have been less optimal powered, since the power calculation was based on the primary outcome PA. However, since we were able to include (and retain) a large number of patients, we expect that underpowering is limited. A post-hoc power calculation for example for fatigue at 3 months (ES = − 0.21), with an alpha of .05, showed to have a power of 0.74.

## Conclusion

The OncoActive intervention was effective at increasing PA in prostate and colorectal cancer patients and survivors both during and after primary cancer treatment. Health-related effects, such as improved fatigue, depression and physical functioning were mainly found in colorectal cancer participants, which also had lower baseline levels. Although long-term maintenance of these effects should be studied, it can be concluded that the intervention provides opportunities to accelerate cancer recovery. In addition, as PA increased in both populations this might have preventive effects for future health status.

Although previous research has suggested that supervised programs result in larger effect sizes, it should also be noted that in view of costs, resources and access, those programs may not be available to everyone [31, 36]. eHealth interventions can be provided at relatively low costs, are more in line with cancer survivors’ preference of home-based PA programs [33, 34] and may also be able to reach those who are not motivated enough to participate in intensive, facility-based programs [35]. Therefore, the results of the current study provide valuable support for the use of the OncoActive intervention to increase PA and improve cancer recovery.

## Additional file

Additional file 1: Line graphs outcomes. Description: line graphs showing the results on the outcome measures over time. (PDF 119 kb)

## Abbreviations

95% CI: 95% confidence intervals
BMI: Body mass index
CIS: Checklist individual strength
EORTC QLQ-C30: European Organization for Research and Treatment of Cancer Quality of Life, Questionnaire – C 30.
ES: Effect size
HADS: Hospital anxiety and depression scale
HRQoL: Health-related quality of life
ICC: Intraclass correlation coefficient
ITT: Intention to treat analysis
MET: Metabolic equivalent
MVPA: Moderate to vigorous physical activity
PA: Physical activity
RCT: Randomized controlled trial
SD: Standard deviation
SQUASH: Short questionnaire to assess health-enhancing physical activity
