# Supplementary material for: Short-term efficacy of a computer-tailored physical activity intervention for prostate and colorectal cancer patients and survivors: a randomized controlled trial
Source: Int J Behav Nutr Phys Act. 2018 Oct 30;15:106. doi: 10.1186/s12966-018-0734-9 (PMC6208119; doi:10.1186/s12966-018-0734-9)

Additional file 1. Line graphs outcomes

Physical Activity Outcomes

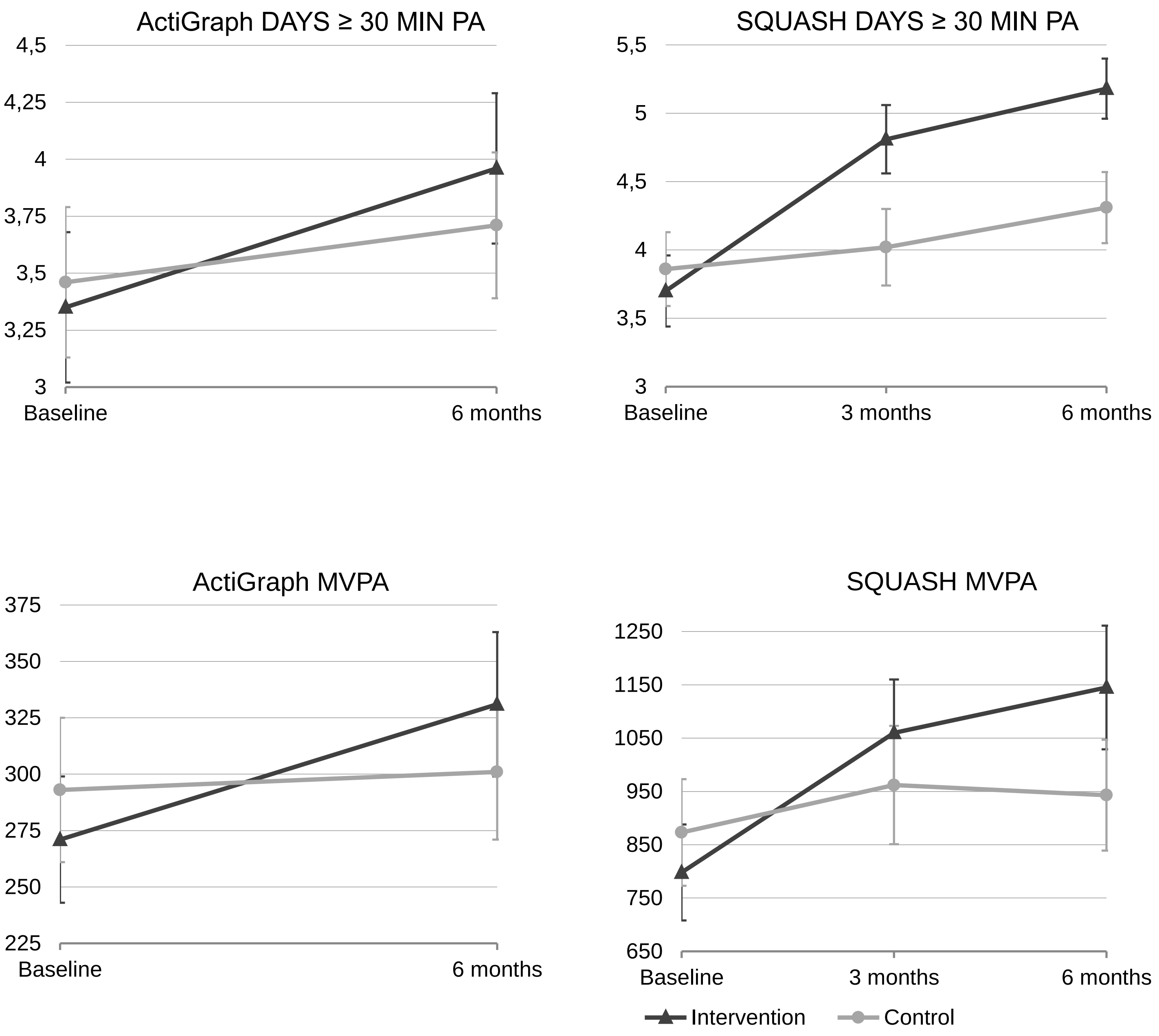

Health-related outcomes

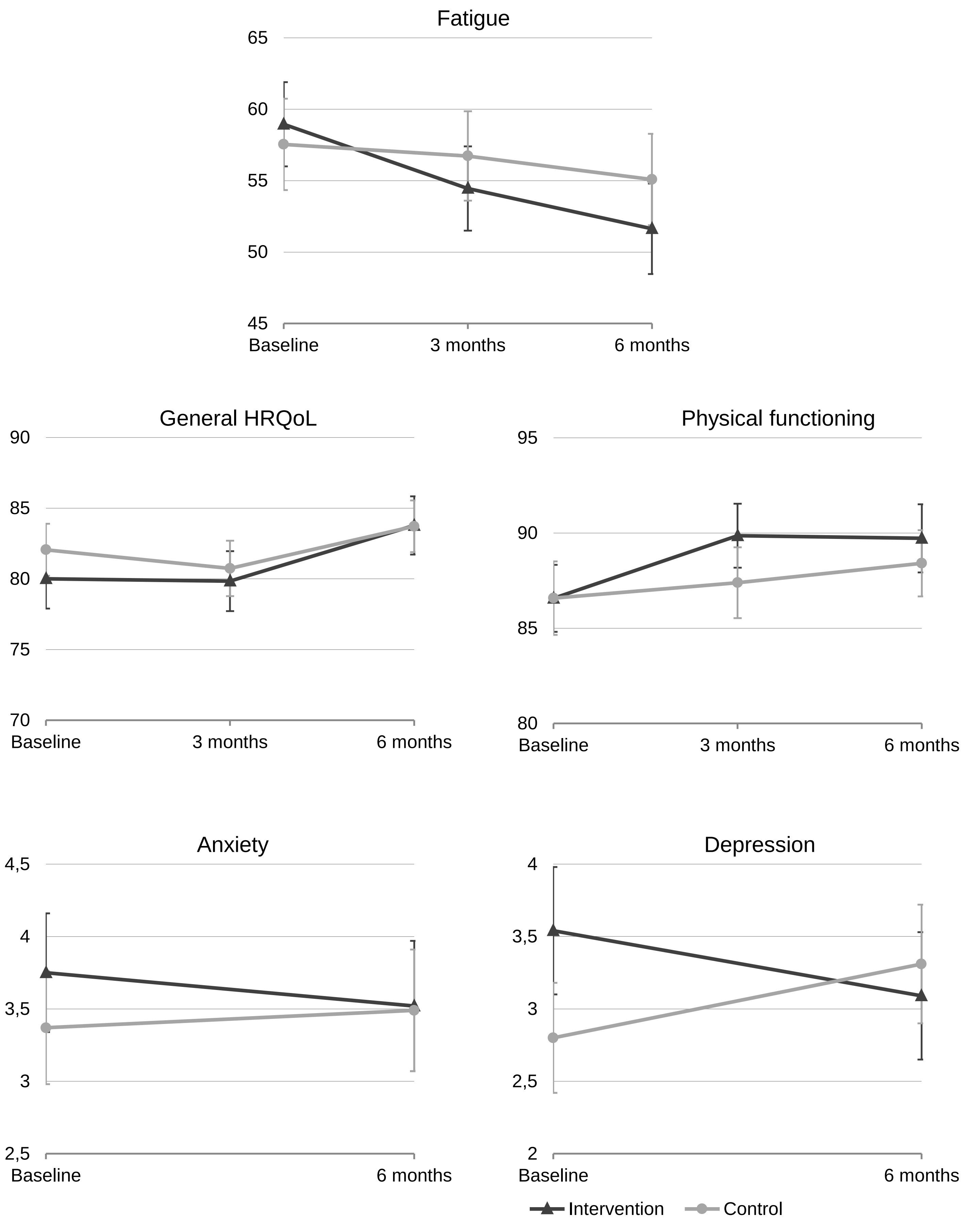

Supplement: Supplementary file 1 — Line graphs outcomes. Description: line graphs showing the results on the outcome measures over time. (PDF 119 kb) [file 12966_2018_734_MOESM1_ESM.pdf]
